# Role of polycystic ovarian syndrome in developing psychological burden in Saudi Arabian females: A case control study

**DOI:** 10.3389/fpubh.2022.999813

**Published:** 2022-11-24

**Authors:** Abdulhakeem S. Alamri, Majid Alhomrani, Walaa F. Alsanie, Mansour Almuqbil, Khawlah M. Alqarni, Saleh M. Alshehri, Osama Abdulaziz, Magdi M. Salih, Bassem M. Raafat, Abdulwahab Alamri, Nasser Fawzan Alomar, Syed Mohammed Basheeruddin Asdaq

**Affiliations:** ^1^Department of Clinical Laboratory Sciences, The Faculty of Applied Medical Sciences, Taif University, Taif, Saudi Arabia; ^2^Centre of Biomedical Sciences Research (CBSR), Deanship of Scientific Research, Taif University, Taif, Saudi Arabia; ^3^Department of Clinical Pharmacy, College of Pharmacy, King Saud University, Riyadh, Saudi Arabia; ^4^Department of Pharmacy, Security Forces Hospital, Riyadh, Saudi Arabia; ^5^Department of Pharmacy, Prince Sultan Military Medical City, Riyadh, Saudi Arabia; ^6^Department of Radiological Sciences, College of Applied Medical Sciences, Taif University, Taif, Saudi Arabia; ^7^Department of Pharmacology and Toxicology, College of Pharmacy, University of Hail, Hail, Saudi Arabia; ^8^Equame Scientific and Research Center, Riyadh, Saudi Arabia; ^9^Department of Pharmacy Practice, College of Pharmacy, AlMaarefa University, Riyadh, Saudi Arabia

**Keywords:** psychological burden, depression, anxiety, stress, hirsutism, polycystic ovary syndrome, education, biological factors

## Abstract

It is well known that polycystic ovarian syndrome (PCOS) may elevate psychological problems in patients, but there is a scarcity of the studies among Saudi Arabian population. This research was designed to investigate the influence of PCOS on the development of psychological load in terms of depression, anxiety, and stress in comparison to normal women who have no PCOS. Further, a correlation of psychological distress in PCOS females was done with their educational level. This is case-control research carried out in one of Riyadh's multispecialty hospitals. In the PCOS patients and control groups (each with 84 samples), samples were collected using convenience sampling and a simple random approach, respectively. The psychological burden was determined using DASS-21. The data obtained were analyzed using SPSS-IBM 25. Most participants (52.9%) were between the ages of 26 and 35 and had a university education (68.4%). A significantly higher percentage of PCOS patients (*P* = 0.001) had irregular menses, hirsutism, infertility, and acne in comparison to the mothers without PCOS. There was a significantly higher possibility of depression (*P* = 0.003), anxiety (*P* = 0.016), and stress (*P* = 0.001) among PCOS patients than in control subjects. Among the psychological domain tested in the study, the risk of developing stress (odds ratio, OR = 8.32) was high when compared to depression (OR = 3.12) and anxiety (OR = 2.127) in PCOS patients. Furthermore, when compared to PCOS females with less education, a significantly lower number of university-educated PCOS females developed depression. The study demonstrates a high prevalence of psychological burden among the PCOS population. Higher education has been shown to help in alleviating depression in PCOS females. Meeting PCOS women's psychological needs will improve their overall health status.

## Introduction

With a prevalence of 6–10%, polycystic ovary syndrome (PCOS) is one of the highly pertinent endocrinal problems reported by women during their childbearing age (1). Anovulation, hyperandrogenism, or polycystic ovaries on ultrasound are three major criteria for the confirmation of PCOS (2). Most of patients' in PCOS will have hirsutism, moderate to severe acne, irregular menses, obesity, and metabolic issues (3). The three main methods of managing PCOS are therapeutic lifestyle changes, drug therapy, and surgery (4).

Several earlier studies have demonstrated a significantly high prevalence rate of psychiatric problems among people all over the world (5–8). Psychiatric diseases are the fourth biggest cause of disease burden in the globe (9). According to a research conducted in Saudi Arabia, the rate of psychiatric diseases ranges from 30 to 46% (10). Unfortunately, the occurance of psychological illness is more prevalence in those who are already burdened with chronic diseases (11). In addition, PCOS women have a higher risk of psychological problems such as depression, anxiety, and stress (12).

According to the World Health Organization, PCOS affects around 3.4% of the world's female population (13). Based on the diagnostic procedures employed in medical settings, the prevalence rate of PCOS varies greatly. However, a recent systematic review found that 27 studies with a mean prevalence of 21.27% of PCOS using various diagnostic criteria (14). The frequency of PCOS varies significantly by geographic area, as well as by racial and ethnic characteristics (15). The 53% prevalence rate indicated by a study is quite concerning because there are no official statistics on the prevalence rate of PCOS in Saudi Arabia (16). Another study carried out in the Saudi Arabian province of Madinah revealed a prevalence of PCOS of 32.5% (17). An online survey-based, more recent Saudi Arabian study found that the prevalence of PCOS was 16% (18). Nonetheless, significant high rate (>36%) of prevalence of psychiatric disorder in the Saudi Arabian community (19) is equally worrying. Although there is a well-known correlation exist between psychological impairments and polycystic ovarian syndrome (20, 21), there are just a few published articles (22) that investigated at this relationship in the Arabian Peninsula, particularly in Saudi Arabia. As a result, it is a source of significant concern, as persistent ignorance may result in the failure to take the required efforts to combat the threat posed by this relationship.

This research was done to investigate the role of polycystic ovarian syndrome on the development of psychological burden such as depression, anxiety, and stress in Saudi Arabian population using a case control study design.

## Materials and methods

### Sampling

This is a case–control research undertaken at one of the multispecialty hospitals of Riyadh, Saudi Arabia. This research was approved by research committee of the AlMaarefa University, Riyadh, Saudi Arabia. The sample size was calculated based on the prevalence report of psychological problems reported earlier (23) in Riyadh which was 28%. Considering an estimated error of 5% and confidence level of 95%, sample size was calculated to be 168 with 84 being in each group (Case group and control group) to get 85% power to the study. The study's participants ranged from age 18 to 50 years. The convenience sample method was utilized to select PCOS cases, while a simple random sampling methodology was employed to recruit control individuals.

### Selection of cases

**Figure 1:**
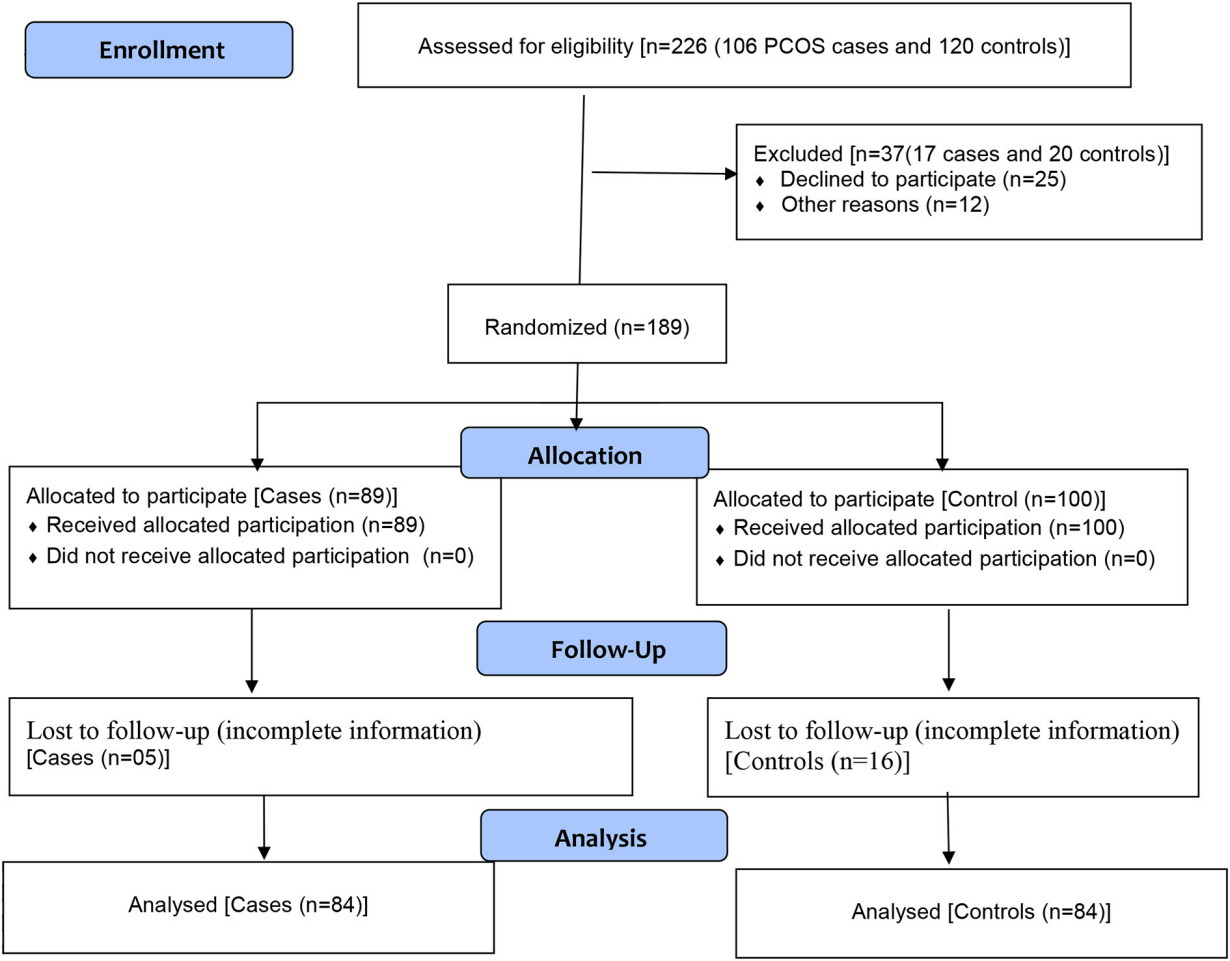
Flow diagram of the study.

The Rotterdam criteria were used to diagnosis the PCOS cases (24). The study excluded women who were pregnant, postmenopausal, those with pituitary disease and cancer. During the study period, 106 PCOS women were identified as appropriate candidates for the study in the outpatient department of the hospital. Only 89 of them accepted to take part in the study (84% response rate). However, because certain essential components of the questionnaire were unfilled in some of the samples, therefore 5 samples were removed and only 84 samples were included for further analysis (Figure 1).

### Selection of controls

During the study period, the females who visited the OB/GYNE departments of the hospital for their regular care were approached for inclusion in the control group. Participants were chosen at random if they were of reproductive age, non-menopausal, non-pregnant, non-PCOS, and did not have any endocrine or metabolic illness that resembled PCOS. 120 eligible patients were reached with the help of a gynecologist. With an 83% response rate, 100 of them accepted to participate in this study. However, due to insufficient data, only 84 controls were included in the analysis (Figure 1).

The questionnaire for this study was prepared with the intention of achieving the desired results (25). The validated Arabic version of the questionnaire was adopted from an earlier study (24). There were three main sections on the questionnaire form. The first section of the questionnaire had items related to sociodemographic characteristics such as age, marital status, education, employment status, and monthly income, while the second section included items related to the clinical signs of PCOS like hirsutism, acne, and irregular menstruation, and the last section had DASS tool (Depression, Anxiety, and Stress Scale-DASS-21) for evaluating the psychological burden of the study participants. This 21-item self-reported screening instrument (26, 27) was adopted to assess psychiatric disorders connected to emotional states like depression, anxiety, and stress (seven questions for each of depression, anxiety, and stress). A 4-point frequency scale (0 to 3) based on the amount of intensity was used to ask the participants to rank their experiences with each item during the previous seven days. The rating of “0” denotes “Did not apply to me at all,” “1” means “Applied to me to some extent,” “2” means “Applied to me to a significant extent,” and “3” means “Applied to me most of the time.” To obtain the final score, the depression, anxiety, and stress scores were summed together and multiplied by two. According to Henry and Crawford (28), the scales were divided into mild, moderate, and severe categories to rate each state's severity. For mild, moderate, and severe depression, the cutoff values were 10, 14, and 21, respectively. For anxiety, the cutoffs were 8, 10, and 15, respectively. Finally, the threshold values for mild, moderate, and severe form were 15, 19, and 26, respectively.

### Statistical analysis

The information gathered was loaded into the IBM SPSS application (version 25). Both descriptive and inferential analysis was done to determine the role of sociodemographic factors on induction of psychological burden in PCOS patients that was compared with data obtained from non-PCOS group. *P-*value < 0.05 was considered significant.

## Results

### Demographic characteristics of the participants

Out of the 168 people chosen for the study, 84 (50%) were PCOS cases and the remaining 84 (50%) were non-PCOS controls. Table 1 shows the details of the participants' distribution based on sociodemographic factors. In both groups, most of the participants (52.9%) were between the ages of 26 and 35 years. Furthermore, in both groups, the bulk of the study samples (>83%) were married. Based on educational background, employment status, and income level, no significant differences were detected among the participants. When compared to control participants, PCOS patients had significantly higher rates of irregular menses, hirsutism, and acne.

**Table 1:** The association between sociodemographic characteristics and PCOS (*n*, %).

**Characteristics**	**PCOS**	**Control**	**Total**	***P*** **-value**
**Age**				0.039^a^
a) ≤ 25 years	18 (21.4)	10 (11.9)	28 (16.6)	
b) 26–35 years	41 (48.8)	48 (58.3)	89 (52.9)	
c) ≥ 36 years	25 (29.7)	26 (30.9)	51 (30.3)	
**Marital status**				0.048^b^
a) Married	77 (91.6)	64 (76.1)	141 (83.9)	
b) Single	08 (9.5)	20 (23.8)	28 (16.6)	
**Education**				0.096^a^
a) University educated	57 (67.8)	58 (69.0)	115 (68.4)	
b) School educated	27 (32.1)	26 (30.9)	53 (31.5)	
**Employment**				0.134^a^
a) Employed	53 (63.0)	59 (70.2)	112 (66.6)	
b) Unemployed	31 (36.9)	25 (29.7)	56 (33.3)	
**Monthly income**				0.061^a^
a) ≥ 10000 SAR	28 (33.3)	38 (45.2)	66 (39.2)	
b) < 10000 SAR	56 (66.6)	46 (54.7)	102 (60.7)	
**Irregular menses**				0.021^b^
a) No	11 (12.2)	43 (90.6)	53 (52.1)	
b) Yes	73 (87.8)	41 (9.4)	114 (47.9)	
**Hirsutism**				0.001^b^
a) No	16 (15.9)	42 (90.6)	58 (53.9)	
b) Yes	68 (84.1)	42 (9.4)	110 (46.1)	
**Presence of acne**				0.001^b^
a) No	45 (53.5)	63 (75)	108 (64.28)	
b) Yes	39 (46.4)	21 (28.57)	60 (35.71)	
**Pregnancy history**				0.046^b^
a) Yes	30 (35.7)	70 (83.3)	100 (59.5)	
b) No	54 (64.2)	14 (16.6)	68 (40.4)	

a Pearson Chi-square test (2-sided);

b Fisher Exact test (2-sided).

### Prevalence of psychological burden

The occurrence of psychological parameters among PCOS cases and control group members is shown in Table 2. Overall, depression, anxiety, and stress were present in 54.16, 55.95, and 61.3% of the subjects, respectively. A significant number of PCOS patients had emotional distress, with 75% having depression (*P* = 0.001), 67.85% having anxiety (*P* = 0.001), and a whopping 85.7 % having stress (*P* = 0.001).

**Table 2:** Prevalence of depression, anxiety, and stress.

**Categories**	**PCOS (84)**	**Control (84)**	**Total (168)**	***P*** -value^*^
**Depression (** ***n*** **)**	63	28	91	0.001
Prevaelnce (%)	75%	33.33%	54.16%	
**Anxiety (** ***n*** **)**	57	37	94	0.001
Prevaelnce (%)	67.85%	44.04%	55.95%	
**Stress (** ***n*** **)**	72	43	115	0.001
Prevalence (%)	85.71%	51.19%	68.45%	

* Pearson Chi-square test (2-sided).

### The intensity of psychological burden

The degree of stress, anxiety, and depression in cases and controls is shown in Table 3. A large percentage of PCOS cases (60.31) reported moderate levels of depression (21.42%) than control patients. Most of the participants in the control group only experienced mild depression (78.57%). Furthermore, the percentage of PCOS patients with high level of anxiety and middle level of stress were more when compared to normal females.

**Table 3:** Level of severity of depression, anxiety, and stress.

**Categories**	**PCOS**	**Control**	**Total**	***P*** -value^*^
**Depression (** ***n*** **)**	63	28	91	0.001
a) Mild	12 (19.04)	22 (78.57)	34 (37.36)	
b) Moderate	38 (60.31)	6 (21.42)	44 (48.35)	
c) Severe	13 (20.63)	0	13 (14.28)	
**Anxiety (** ***n*** **)**	57	37	94	0.001
a) Mild	12 (21.05)	7 (18.91)	19 (20.21)	
b) Moderate	21 (36.84)	30 (81.08)	51 (54.25)	
c) Severe	24 (42.10)	0	24 (25.53)	
**Stress (** ***n*** **)**	72	43	115	0.001
a) Mild	20 (27.77)	26 (60.46)	46 (40)	
b) Moderate	39 (54.16)	17 (39.53)	56 (48.69)	
c) Severe	13 (18.05)	0	13 (11.30)	

* Pearson Chi-square test (2-sided).

### The analysis of factors that influences psychological burden

The logistic regression analysis of psychological parameters in PCOS women is shown in Table 4. When comparing PCOS cases to control participants, the probabilities of experiencing psychological distress, such as depression (*P* = 0.003), anxiety (*P* = 0.016), and stress (*P* = 0.001), were considerably greater in PCOS cases. Additionally, participants with low level of education showed significantly (*P* = 0.018) higher proportion of depression status compared to university educated surveyors. However, no significant impact was noticed on anxiety and stress due to variation in educational level. Further, significantly higher number of unmarried females exhibited depression (*P* = 0.011) and stress (*P* = 0.045) compared to married ones.

**Table 4:** Logistic regression analysis of depression, anxiety, and stress.

**Categories**	**Odds ratio**	**Confidence interval**		***P*** **-value**
		**(95%)**		
		**Lower**	**Upper**	
**Depression**
a) PCOS status	3.123	1.122	6.32	0.003
b) Educational level	1.328	0.989	2.98	0.018
c) Marital status	1.291	0.876	1.99	0.011
**Anxiety**
a) PCOS status	2.127	1.111	4.054	0.016
b) Educational level	1.120	0.659	2.132	0.244
c) Marital status	1.111	0.543	1.876	0.231
**Stress**
a) PCOS status	8.322	5.654	18.118	0.001
b) Educational level	1.169	0.598	2.032	0.265
c) Marital status	1.876	1.324	2.674	0.045

## Discussion

This study used a case control technique to analyze the impact of sociodemographic features and signs and symptoms of PCOS in confirmed PCOS cases in comparison to patients who have not been diagnosed with PCOS. Further, the influence of the presence of PCOS on psychological burdens such as depression, anxiety, and stress were evaluated among the Saudi Arabian population residing in Riyadh, Saudi Arabia.

In the PCOS cases, there was a higher susceptibility to irregularities in menses, hirsutism, and infertility than acne, as indicated in the results section (Table 1). Our findings are consistent with previous studies in PCOS women in the Arabian Peninsula (22) and South Asia (27). In Oman (22), high indices of hirsutism and anovulation were found, while in South Asia (27), secondary infertility, oligomenorrhea, and hirsutism were shown to be dominating features in PCOS cases. Saudi Arabians and Omanians have a shared culture and religion, and there are significant similarities between them (28). As a result, it is probable that Saudi Arabians have identical PCOS symptoms. We noticed a link between hirsutism and psychological issues like stress, depression, and anxiety (data not included). It is crucial to remember that PCOS with hirsutism is frequently thought of as a variant of PCOS with high androgen levels. However, hirsutism can exist in women with low androgen levels because it results from an interplay between androgens and the sensitivity of the female hair follicle to androgens. The gold standard diagnostic for hirsutism, the Ferriman-Gallwey score, was found to be strongly correlated with the testosterone and estrogen precursors androstenedione and dehydroepiandrosterone sulfate, but not with other biochemical indicators of hyperandrogenism in women with PCOS. Further data is required to determine if the links between hirsutism and psychological issues observed in this study are mediated by increased androgen levels (29). Regardless of the explanation, having unwanted hair can make people shy away from social situations and feel self-conscious. This has a negative impact on one's mental health and social behavior. Over time, these clinical symptoms frequently turn into psychological distress in the form of stress, anxiety, and depression. The results of this study on the relationship between mental distress and hirsutism are like those of an earlier study (30). Even a thorough search of the literature revealed no recent studies in Saudi Arabia that used the DASS-21 to investigate the impact of PCOS on psychological problems other than a study done by us earlier (24). DASS-21, unlike other symptom measures, allows us to assess not only the presence but also the severity of three common psychological illnesses: depression, anxiety, and stress. Overall, moderate levels of stress were more widespread (Table 3) than depression and anxiety in this study, which is consistent with previous studies (31). The experience of negative emotions that may occur owing to abnormal physiological, biochemical, cognitive, and behavioral alterations is the source of stress (32).

In the literature, there is a well-established link between depression and several chronic conditions (33). According to a study conducted in Saudi Arabia, the general population of Saudi Arabia has a depression prevalence rate of 28.5% (34). Women are more susceptible to depression than men, and the existence of gynecological disorders has been shown to act as a stimulant for depression (34). In our study, depression was shown to be more common in PCOS individuals than in normal women in our study (70 vs. 47%). Furthermore, the majority of PCOS women reported moderate depression, whereas the control group reported mild levels of depression. Serotonin (5-HT), dopamine (DA), gamma-aminobutyric acid (GABA), and acetylcholine (Ach), which act as inhibitory neurotransmitters, are reduced in PCOS. The principal stimulants of GnRH and LH, glutamate, are elevated in PCOS-related diseases, and these neurotransmitter changes may contribute to the pathophysiology of depression in PCOS (35). There are other potential contributing factors, including obesity, insulin resistance, hyperandrogenism, inflammation, and infertility, but the precise mechanisms causing the greater prevalence of depressive symptoms in PCOS-positive women are not yet known (36). The mood swings in our PCOS group patients could be caused by PCOS-related hyperandrogenism, which can disrupt monoamine balance and contribute to depression in PCOS patients (37). Our findings are consistent with previous reports of PCOS women having a higher incidence of depression (38).

Despite the fact that our research met its objectives, it has several limitations. First, a self-reported questionnaire served as the basis for the outcome. As a result, the questions may be misunderstood or reported with bias. Second, our questionnaire includes statements regarding participants' feelings and capacity to accomplish certain tasks over the last 1 week, which relies on the ability to recall, which some participants lack. In addition, despite receiving consent from hospital administration, it might be challenging to reach hospital patients, especially those in the gynecology and obstetrics departments, due to cultural concerns. This has resulted in a modest sample collection. To further corroborate our findings, studies with bigger sample sizes may be required. It is difficult to estimate the prevalence rate with this small sample. Further, there is lack of homogeneity of sampling between case and control groups in terms of the age and marital status, which could influence the development of psychological stress instead of PCOS per se. Also, it is unclear whether the depression found in PCOS was simply related to PCOS or other concurrent causes; a more thorough data sheet would have provided an answer. The incidence of depression is often higher in people of reproductive age. Finally, the study on PCOS predictors is incomplete because there may be additional predictors besides those evaluated in this study.

## Conclusion

The findings show that the PCOS group has a significant frequency of psychological distress. Stress was the dominating psychological distress compared to depression and anxiety among patients with PCOS. This study's findings may be useful to healthcare providers, especially those who work with PCOS women. It also introduces the potential of PCOS patients being required to undergo mental health screening.

## Data availability statement

The original contributions presented in the study are included in the article/supplementary material, further inquiries can be directed to the corresponding author.

## Ethics statement

The studies involving human participants were reviewed and approved by Research Committee, AlMaarefa University. The patients/participants provided their written informed consent to participate in this study.

## Author contributions

Under the supervision of SMBA, ASA, MAlh, and WA carried out the research methodology. OA, MS, NA, KA, and BR were responsible for formal analysis of the work. WA, SMA, ASA, MAlh, MAlm, and AA participated in writing original draft of the manuscript. ASA administered the project. SMBA was instrumental in review and editing of the manuscript. All authors contributed to the article and approved the submitted version.

## Funding

The authors extend their appreciation to the Deputyship of Research and Innovation, Ministry of Education in Saudi Arabia for funding this research work through the project number 1-442-56.

## Conflict of interest

The authors declare that the research was conducted in the absence of any commercial or financial relationships that could be construed as a potential conflict of interest.

## Publisher's note

All claims expressed in this article are solely those of the authors and do not necessarily represent those of their affiliated organizations, or those of the publisher, the editors and the reviewers. Any product that may be evaluated in this article, or claim that may be made by its manufacturer, is not guaranteed or endorsed by the publisher.
